# Categorization of Cytochrome P4502D6 Activity Score by Urinary Amphetamine/Methamphetamine Ratios

**DOI:** 10.3390/metabo12121174

**Published:** 2022-11-25

**Authors:** Jatuporn Chaichana, Manee Khamenkhetkarn, Thanapat Sastraruji, Tawachai Monum, Timothy E. O’Brien, Yutti Amornlertwatana, Churdsak Jaikang

**Affiliations:** 1 Toxicology Section, Regional Medical Science Center 1 Chiang Mai 191 Tumbon Don Keaw, Ampher Mae Rim, Chiang Mai 50180, Thailand; 2 Faculty of Dentistry, Chiang Mai University, Chiang Mai 50200, Thailand; 3 Department of Forensic Medicine, Faculty of Medicine, Chiang Mai University, Chiang Mai 50200, Thailand; 4 Department of Mathematics and Statistics, Loyola University Chicago, 1032 W.Sheridan Road, Chicago, IL 60660-1537, USA

**Keywords:** CYP2D6, activity score, metabolic ratio, methamphetamine, amphetamine

## Abstract

Methamphetamine (MA) level in urine has been used for judgment in MA consumption. Metabolism and intoxication of MA are correlated with the activity of cytochrome P450 2D6 (CYP2D6). The activity score (AS) is a potential tool for predicting exposure and personalized dose of drugs metabolized by CYP2D6. Prediction of the CYP2D6 activity score might be described as MA intoxication. The objective of this study was to categorize the CYP2D6 activity score using the urinary amphetamine (AM)/MA ratio. Urine samples (n = 23,258) were collected. The levels of MA and AM were determined by a gas chromatography–nitrogen–phosphorus detector. The log AS was calculated by an AM/MA ratio and classified into four groups following the percentile position: lower than the 2.5th, the 2.5th–the 50th, the 50th–97.5th, and greater than the 97.5th percentile, respectively. The AS value for males presented was less than 0.024, 0.024–0.141, 0.141–0.836, and greater than 0.836. Meanwhile, the AS values were revealed to be lower than 0.023, 0.023–0.148, 0.148–0.850, and higher than 0.850 for females. The AS value of CYP2D6 can be applied to describe the toxicity of MA in forensic crime scenes and relapse behavior.

## 1. Introduction

Cytochrome P450 2D6 (CYP2D6) is the second largest xenobiotic-metabolizing enzyme in the phase I metabolic pathway and is a major expression in the liver [1,2]. CYP2D6 transforms a functional group to the xenobiotic molecules, including methamphetamine (MA), dextromethorphan, bufuralol, debrisoquine, risperidone, and venlafaxine to highly polar properties [3,4,5,6]. To date, over 140 allelic variants have been designated by the pharmacogenetic variation consortium and are involved in CYP2D6 activity [7]. The high polymorphism of the *CYP2D6* gene is an important factor explaining the wide range of CYP2D6 activity.

The activity score (AS) is a tool for reflecting enzyme activity related to personalizing genes and phenotypes [5]. The activity score is calculated by the product/substrate ratio in various biological samples [8]. Some specific substrates, including codeine, risperidone [6], dextromethorphan [5,6], and methamphetamine (MA) [9] are assigned for the AS. Improvement and development of the AS system to predict CYP2D6 activity may help more fully understand the intrinsic factors that affect drug metabolism.

Dextromethorphan has been used as a substrate for estimating CYP2D6 AS and the dexthrophan/dextromethorphan ratio has been determined in urine, plasma, serum, and whole blood [10,11]. In some studies, risperidone has been applied for the CYP2D6 AS value, and 9-hydroxyrisperidone and its substrate have been determined in plasma [12]. Variations of substrates and biological samples affect the CYP2D6 AS value [13,14]. Suitable specific substrates for CYP2D6 AS evaluation should be considered, especially drug-related diseases and environmental factors.

**Figure 1:**
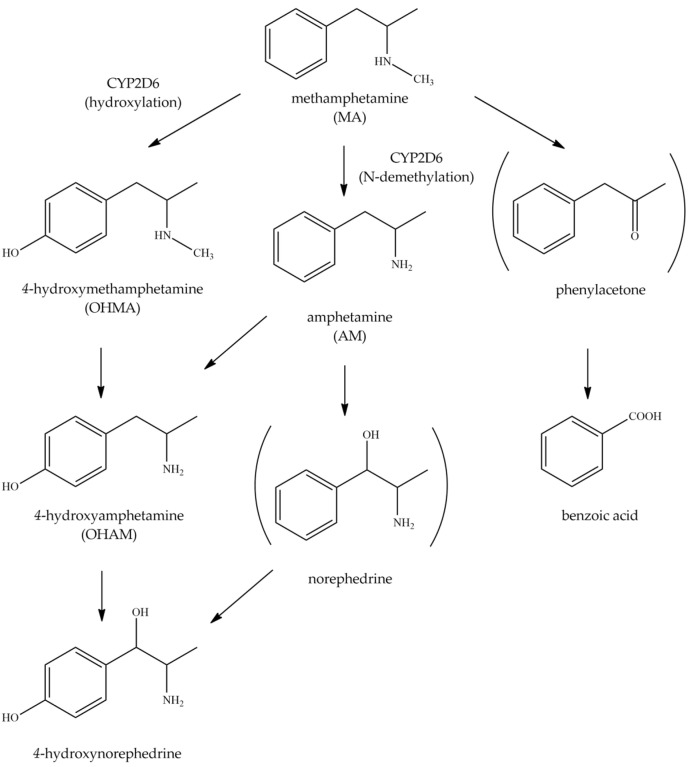
The metabolic pathway of methamphetamine [23].

Many illicit drugs including methamphetamine (MA), morphine, and heroin are smuggled from neighboring countries into Northern Thailand and have impacted public health and national security [15]. MA is a psychostimulant drug and is the most illicit drug problem. MA toxics in the central nervous system increase dopamine, serotonin, and gamma-aminobutyric acid (GABA) levels [16,17,18,19,20]. MA is the most consistently associated with impaired executive functions, attention, information processing speed, episodic memory, verbal fluency, and motor skills relating to criminal crimes. CYP2D6 is a major enzyme for MA catabolism via 4-hydroxylation (8–11%) and *N*-demethylation (2–7%) reaction to provide 4-hydroxymetamphetamine and amphetamine (AM), respectively [21,22]. The 4-hydroxymethamaphetamine and 4-hydroxyamphetamine are inactive forms but AM is an active metabolite of MA. The mechanism of methamphetamine is shown in Figure 1.

In Thailand, urine samples from hospitals, police stations, academics, and industries have been sent to evaluate MA exposure. Chronic MA addiction, and death from MA intoxication at low doses are still problems in Thailand and cannot be described with MA levels in both urine and blood samples. The CYP2D6 phenotype affects metabolism and individual MA response [6] and should be evaluated to understand the problems related to CYP2D6 activity. The CYP2D6 AS model specific to MA exposure for the Northern Thai population needs to be developed. The aim of this study was to characterize the CYP2D6 activity scores by using urine MA samples.

## 2. Materials and Methods

### 2.1. Chemicals and Materials

Certified reference standards of MA, AM, phentermine, phenylpropanolamine, pseudoephedrine, ephedrine, 3- and 4-methylenedioxyamphetamine (MDA), 3- and 4-methylenedioxymethamphetamine (MDMA), and 3- and 4-methylenedioxy-N-ethyl-amphetamine (MDE) were purchased from Lipomed (Arlesheim, Basel-Stadt, Switzerland). Potassium carbonate and sodium azide were purchased from Merck (Darmstadt, Germany). Blank human urines were prepared by six healthy volunteers. The solid phase microextraction (SPME) fiber, coated with 100 mm of polydimethylsiloxane (PDMS), was purchased from Agilent (Santa Clara, CA, USA).

### 2.2. Standard Solutions and Quality Controls Preparation

Stock standard solutions of MA, AM, and phentermine (internal standard) were prepared at 1 g/L in methanol. The standard and quality control solutions were prepared from the stock solutions and dissolved in the drug-free human urine samples. A calibration curve was prepared with various concentrations of MA and AM (0.5–5.0 μg/mL). Three quality controls were prepared (0.75, 1.5, and 3.0 μg/mL).

### 2.3. Method Validation

Method validation was performed using a bioanalytical validation guideline [24]. Selectivity was proved by the six different sources of human urine. Mixed standards MA and AM determined the linearity of MA and AM at a range of 0.1–10.0 μg/mL. Accuracy and precision were analyzed by the within- and between- run of the quality controls. The limit of detection, the limit of quantification, the correlation coefficient (r^2^), the between-run of accuracy and precision values, the relative difference (%RD), and the coefficient of variation (%CV) followed the bioanalytical validation guideline.

### 2.4. SPME GC–NPD Condition

The gas chromatography–nitrogen–phosphorous detector (GC–NPD; Agilent 8890 GC system) is equipped with an autosampler system (Zwingen, Basel-Landschaft, Switzerland). The GC column was an Rtx-35 Amine (30 m × 0.25 mm, i.d., 0.5 μm film thickness). The temperature of the GC–NPD condition was started at 80 °C for 0.5 min, then increased to 150 °C with a temperature rate of 40 °C/min. After that, the temperature was increased to 200 °C with a rate of 15 °C/min and finally held at 200 °C for 0.5 min. The volatile urine sample was adsorbed by the SMPE fiber for 5 min and subsequently desorbed into the GC injector port at 220 °C for 30 s. The split mode (a split ratio of 10:1) was used and the detector temperature was 220 °C. Helium was used as carrier gas and the flow rate was set at 1.5 mL/min. Acceptable retention times of the target chemicals are considered to be within a time window of ±0.1 min.

### 2.5. Urine Samples Collection

Urine samples (n = 23,258) were collected only from the Northern Thai population, indicated by a Thai national identification number (ID) card, and preserved by sodium azide. The study protocol was approved by the Research Ethics Committee Faculty of Medicine, Chiang Mai University (FOR: 2565-09056). Urine samples that found only MA or AM, the level of MA and AM less than 0.5 μg/mL, the CYP2D6 inhibitors (amitryptyline, quinidine, and fluoetine), and the CYP2D6 inducers (dexamethasone and rifampin) were excluded in this study.

### 2.6. Determination of Methamphetamine and Amphetamine Levels

The urine sample (500 µL), phentermine solution (an internal standard), and 5Mpotassium carbonate solution were added into a vial. The solution was mixed for 30 s before analysis with the validated SPME GC–NPD method.

### 2.7. Estimation of a Relative Location of the CYP2D6 Activity Score

The activity score was calculated using the value of AM/MA ratio. The AM/MA ratios were transformed using a logarithm, two-tailed at a 95% confidence interval, and categorized for the percentile position. Location data measurement of the log AS was set into an equal-sized range of values (the 2.5th, 50th, and 97.5th percentiles).

### 2.8. Statistical Analysis

The descriptive statistics were presented as mean ± standard deviation (SD). The normality was tested with the Kolmogorov–Smirnov test. The mean value of log AS in each of the phenotypes was compared by one-way ANOVA following LSD. The Statistical Package for the Social Sciences (SPSS) version 22 (SPSS Software, SPSS Inc., Chicago, IL, USA) was used for analysis. The statistical significance is reported as *p* < 0.05.

## 3. Results

### 3.1. Method Validation

**Figure 2:**
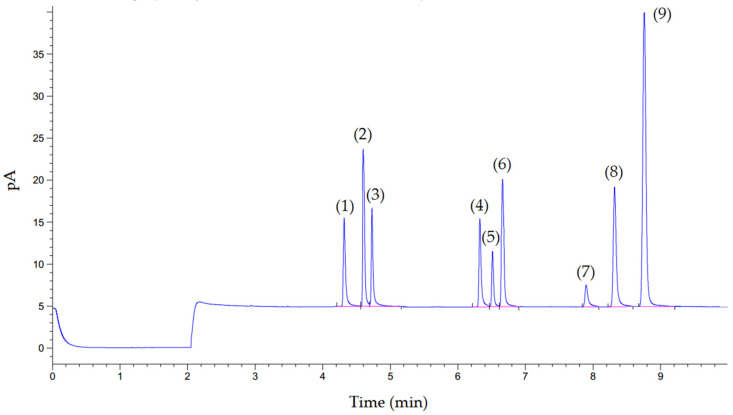
Chromatogram of the standards in pooled urine samples; amphetamine (1), phentermine (2), methamphetamine (3), phenylpropanolamine (4), pseudoephedrine (5), ephedrine (6), MDA (7), MDMA (8), and MDE (9).

Ephedrine, pseudoephedrine, phenylpropanolamine, MDMA, MDA, MDE, and other substances did not interrupt MA, AM, or phentermine determination. The chromatogram of all compounds is shown in Figure 2. The total run time was 10 min. The linearity range of MA and AM was assessed by using eight concentrations of MA and AM solution mixtures (0.3–7.5 μg/mL). The correlation coefficient (r^2^) of MA and AM was 0.995 (r^2^ > 0.98), indicating good linearity. The accuracy of linearity was evaluated by a back-calculated concentration of the calibration standards. All the standard concentrations presented did not exceed ±15%. The limit of detection (LOD) and the limit of quantification (LOQ) of both urinary MA and AM were 0.3 and 0.5 μg/mL, respectively. Results of the within- and between-run accuracy and precision are presented in Table 1. All parameters were accepted under the criteria of the bioanalytical validation guideline. The sample stability was tested, and the results showed that the concentration of MA and AM were stable after storage for 1 year at −70 °C (a decrease of less than 10%).

### 3.2. Urinary Methamphetamine and Amphetamine Data

Ephedrine, pseudoephedrine, phenylpropanolamine, MDMA, MDA, and MDE were not found in all the selected samples. In this study, the urine samples of males (n = 15,656) and females (n = 1484) were collected from many institutes, including police stations, academics, hospitals, factories, and penitentiaries. Levels of MA and AM in urine samples of males and females are shown in Table 2. The mean value of MA and AM levels in the samples of males and females did not differ.

### 3.3. Categorization of the CYP2D6 Activity Score in the Population

**Figure 3:**
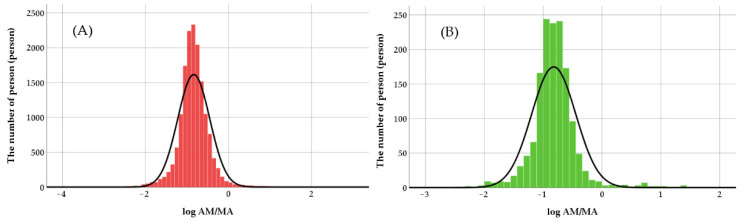
Histograms show a distribution of log (AM/MA) in urine samples of males (**A**) and females (**B**).

The normality distribution of log (AM/MA) value for male and female were tested with the Kolmogorov–Smirnov test. The data presented normal distribution (*p* < 0.001) in both males and females (Figure 3). Under normal distribution of log (AM/MA), the CYP2D6 activity score was classified into four groups according to the relative location; less than the 2.5th, the 2.5th–50th, the 50th–97.5th, and greater than the 97.5th percentile, respectively. The AS value for males presented was less than 0.024, 0.024–0.141, 0.141–0.836, and greater than 0.836. For females, the values were revealed to be less than 0.023, 0.023–0.148, 0.148–0.850, and greater than 0.850, respectively, and the results are shown in Table 3 and Table 4.

## 4. Discussion

The best of our knowledge showed that the CYP2D6 activity scores differed by gender. The AS value of males presented was less than 0.024, 0.024–0.141, 0.141–0.836, and greater than 0.836. For females, the values were revealed to be below 0.023, 0.023–0.148, 0.148–0.850, and exceed 0.850 for lower than the 2.5th, between the 2.5th and the 50th, between the 50th and 97.5th and greater than the 97.5th percentile, respectively.

CYP2D6 is an essential member of the CYP superfamily constituting about 2–4% of the total hepatic CYP contents [25]. Currently, many different drugs have been recommended for CYP2D6 drug clinical guidelines including codeine, tamoxifen, venlafaxine, doxepin, imipramine, and nortriptyline [26]. There are many AS values that are specific and based on the substrates. The activity score is an effective tool to predict personalized metabolizer status from gene information [27]. CYP2D6 pharmacogenomic guidelines use the AS to avoid harmful doses in patients. Many endogenous and exogenous substances are metabolized by CYP2D6. The metabolic ratio and the AS have the potential tools to simplify CYP2D6 phenotypes [5,12,28,29].

CYP2D6 phenotypes are classified into four groups including poor metabolizers (PMs), intermediate metabolizers (IMs), extensive metabolizers (EMs), and ultra-rapid metabolizers (UMs) [5]. The PM phenotype lacks two functional alleles, but the IM phenotype has one reduced-activity allele and one non-functional allele or reduced-activity allele. EM individuals have one or two functional alleles and the UM phenotype is linked with gene duplications of functional alleles [30].

The concentration ratio of product/substrate represents the enzyme activity. Morphine/codeine [31], 9-hydrorisperidone/risperidone, and dehydroaripiprazole/aripiprazole [6] are used for calculating the AS of CYP2D6. However, the concentration of substrate/product, including dextromethorphan/dexthrophan [32], is presented as a metabolic ratio. In this study, the AM/MA ratio was used for the estimation of CYP2D6 phenotypes. The urinary MA level in males and females was 29.38 ± 52.76 and 26.47 ± 31.85 µg/mL, respectively. The AM level in males and females was 4.34 ± 16.29 and 4.35 ± 14.78 µg/mL, respectively. The mean ratio of AM/MA is reflected in CYP2D6 activity.

MA addiction is a chronic and complicated problem in Northern Thailand. Methamphetamine tablets have been composed of only methamphetamine. The judgment process and the government occasionally need more scientific data, especially genotypes and phenotypes of CYP2D6 to describe and solve the problems. The AS values of CYP2D6 are varied and specified to the substrates and source of specimens. Specific CYP2D6 substrates should be considered under conditions including safety, utility, budget, and complexity of determination processes. The determination of MA and AM levels in urine is a routine laboratory for the judgment of MA exposure under Thai law. Under the condition, dextromethorphan, risperidone, and metoprolol are not suitable substrates for the identification of CYP2D6 phenotypes. MA is directly metabolized via CYP2D6. The levels of MA and its metabolites were determined and used to monitor the MA intoxication via the CYP2D6 pathway [30]. Then, MA is the most appropriate substrate for reflecting CYP2D6 activity.

Usually, oral, intravenous injection, smoking, inhalation, and snorting are routes of MA administration [33]. In this study area, vaporized MA inhalation is a major route of MA exposure. The route of MA exposure is a significant factor in absorption, distribution, excretion, metabolism, and toxicity [34], resulting in variations in blood and urine MA levels.

In the previous study, AM/4-hydroxyamphetamine was used for CYP2D6 phenotypes classification [9]. Commonly, the AS value presents more than zero. The urinary metabolic ratio (AM/4-hydroxyamphetamine) of EM, IM, and PM is presented in the range of 0.95–13.11, 15.22–24.96, and 28.57–100, respectively. Our results showed that the AS value of males presented was less than 0.024, 0.024–0.141, 0.141–0.836, and greater than 0.836. For females, the AS value were revealed to be less than 0.023, 0.023–0.148, 0.148–0.850, and greater than 0.850 for PM, IM, EM, and UM, respectively. For the classification of CYP2D6 metabolizers, the values from different models (substrate/product or product/substrate) should be considered during evaluation.

At the relative position of the data, in which the value is less than the 2.5th percentile, the ratio of AM/MA of the male was lower than the female about four times. The AM/MA ratio in this position indicates that it is the lowest velocity of demethylation reaction via CYP2D6 enzyme compared with the other positions. The lower AM/MA ratio might represent PM metabolizers. The low ability of MA metabolism leads to a prolonged half-life and more MA intoxication.

Meanwhile, the ratio of AM/MA of the males at the position greater than the 97.5th percentile showed higher than females about 20 times indicating rapid MA metabolism. Under high metabolism, MA is fast changed to AM leading to short haft-life in the body. This position (greater than the 97.5th percentile) might represent to UM metabolizer. Then, people in a position greater than the 97.5th percentile might consume MA at a higher dose and more frequency than the other groups.

CYP2D6 activity is influenced by many non-genetic factors, especially drugs and foods. Before the study, all urine samples have screened the drugs that can inhibit or induce CYP2D6 activity such as dextromethorphan, nortriptyline, eliglustat, fluoxetine, paroxetine, and bupropion [27,35]. In addition, phytochemicals and associated chemicals contained in food have also influenced CYP2D6, including sesamin [36], curcumin, and the botanical herb, goldenseal [37]. Gender and sex hormones [38] are major factors in CYP2D6 activity.

Exposure to methamphetamine is still a significant problem in Northern Thailand and cannot solve this problem. CYP2D6 is highly related to MA metabolism and needs to genotype identification. However, identification of the CYP2D6 genotype must be used at a high cost. The AM/MA ratio might be an appropriate biomarker for evaluating CYP2D6 activity. The utility of the AM/MA value might be described as MA metabolism in the addicts and MA intoxication (at a low dose) in forensic medicine. Our study had several limitations. Firstly, time and dose of exposure to MA cannot be provided. Secondly, smoking, alcoholism, diseases, and oral contraceptives should be evaluated.

## 5. Conclusions

In this study, the *N*-demethylation of MA reaction is represented as CYP2D6 activity. The AS of CYP2D6 was calculated by the AM/MA ratio. The AS value of males presented was less than 0.024, 0.024–0.141, 0.141–0.836, and greater than 0.836. Meanwhile, the AS values were revealed to be lower than 0.023, 0.023–0.148, 0.148–0.850, and higher than 0.850 in females at the percentile position; lower than the 2.5th, the 2.5th–the 50th, the 50th–97.5th, and greater than the 97.5th percentile, respectively. The urinary AM/MA ratio might be helpful for describing MA metabolism in addicts and MA intoxication in forensic medicine.

## Figures and Tables

**Table 1:** The results of the within-run and the between-run accuracy and precision.

Quality Controls Concentration (μg/mL)	Concentration Mean ± SD (μg/mL)		Precision CV (%)		Accuracy RD (%)	
	MA	AM	MA	AM	MA	AM
Within-run (n = 5)						
Low (0.75)	0.82 ± 0.01	0.80 ± 0.01	0.8	0.8	8.4	9.8
Medium (1.50)	1.52 ± 0.04	1.48 ± 0.05	2.0	3.2	−0.3	−0.3
High (3.00)	3.02 ± 0.05	3.01 ± 0.06	3.5	1.7	−0.4	0.0
Between-run (n = 15)						
Low (0.75)	0.84 ± 0.02	0.81 ± 0.01	2.3	1.7	11.3	11.3
Medium (1.50)	1.56 ± 0.05	1.50 ± 0.07	3.1	4.8	2.4	1.4
High (3.00)	3.12 ± 0.13	3.10 ± 0.17	4.3	5.6	1.6	4.5

**Table 2:** Methamphetamine and amphetamine levels in urine samples.

Concentration (µg/mL)	Male (n = 15,656)	Female (n = 1484)
Methamphetamine	29.38 ± 52.76	26.55 ± 31.95
99% CI of MA	28.29–30.46	24.42–28.69
Min-Max value of MA	0.01–2603.73	0.12–351.19
Amphetamine	4.34 ± 16.34	4.35 ± 14.78
99% CI of AM	4.01–4.68	3.36–5.34
Min-Max value of AM	0.02–677.14	0.05–305.31

**Table 3:** Categorization of the CYP2D6 activity score using log (AM/MA) values from male urine samples.

Positions	CYP2D6 Activity Score (×10^−1^)	Number	99% CI for Mean Activity Score	Range of CYP2D6 Activity Score
<2.5th	<0.24	393	0.012–0.013	2.51 × 10^−4^–2.42 × 10^−2^
2.5th–50th	0.24–1.41	7437	0.087–0.088	2.42 × 10^−2^–0.14
50th–97.5th	1.41–8.36	7435	0.232–0.236	0.14–0.84
>97.5th	>8.36	391	2.443–3.090	0.84–530.36

**Table 4:** Categorization of the CYP2D6 activity score using log (AM/MA) value from female urine samples.

Positions	CYP2D6 Activity Score (×10^−1^)	Number	99% CI for Mean Activity Score	Range of CYP2D6 Activity Score
<2.5th	<0.23	37	0.011–0.015	2.96 × 10^−3^–2.23 × 10^−2^
2.5th–50th	0.23–1.48	705	0.091–0.096	2.58 × 10^−2^–0.15
50th–97.5th	1.48–8.50	705	0.228–0.241	0.15–0.84
>97.5th	>8.50	37	2.539–4.825	0.85–25.89

## Data Availability

The data presented in this study are available in the main article.
